# Magnetic Alginate/Chitosan Nanoparticles for Targeted Delivery of Curcumin into Human Breast Cancer Cells

**DOI:** 10.3390/nano8110907

**Published:** 2018-11-05

**Authors:** Wenxing Song, Xing Su, David Alexander Gregory, Wei Li, Zhiqiang Cai, Xiubo Zhao

**Affiliations:** 1 Department of Chemical and Biological Engineering, University of Sheffield, Sheffield S1 3JD, UK; wsong4@sheffield.ac.uk (W.S.); d.a.gregory@sheffield.ac.uk (D.A.G.); 2 School of Pharmaceutical Engineering and Life Science, Changzhou University, Changzhou 213164, China; zhqcai@cczu.edu.cn; 3 Department of Materials Science and Engineering, University of Sheffield, Sheffield S1 3JD, UK; whitesnowleopard@126.com; 4 Department of Electronic and Electrical Engineering, University of Sheffield, Sheffield S3 7HQ, UK; WLi27@sheffield.ac.uk

**Keywords:** alginate, chitosan, layer-by-layer, magnetic nanoparticles, drug delivery, cancer, curcumin

## Abstract

Curcumin is a promising anti-cancer drug, but its applications in cancer therapy are limited, due to its poor solubility, short half-life and low bioavailability. In this study, curcumin loaded magnetic alginate/chitosan nanoparticles were fabricated to improve the bioavailability, uptake efficiency and cytotoxicity of curcumin to Human Caucasian Breast Adenocarcinoma cells (MDA-MB-231). Alginate and chitosan were deposited on Fe_3_O_4_ magnetic nanoparticles based on their electrostatic properties. The nanoparticle size ranged from 120–200 nm, within the optimum range for drug delivery. Controllable and sustained release of curcumin was obtained by altering the number of chitosan and alginate layers on the nanoparticles. Confocal fluorescence microscopy results showed that targeted delivery of curcumin with the aid of a magnetic field was achieved. The fluorescence-activated cell sorting (FACS) assay indicated that MDA-MB-231 cells treated with curcumin loaded nanoparticles had a 3–6 fold uptake efficiency to those treated with free curcumin. The 3-(4,5-Dimethylthiazol-2-yl)-2,5-Diphenyltetrazolium Bromide (MTT) assay indicated that the curcumin loaded nanoparticles exhibited significantly higher cytotoxicity towards MDA-MB-231 cells than HDF cells. The sustained release profiles, enhanced uptake efficiency and cytotoxicity to cancer cells, as well as directed targeting make MACPs promising candidates for cancer therapy.

## 1. Introduction

Curcumin (CUR) is a yellow, hydrophobic, polyphenolic compound of turmeric that is extracted from the rhizomes of *Curcuma longa*, which are widely cultivated in Asian countries, such as India and China, and have been historically used as a spice [1]. CUR is generally recognised as safe (GRAS) by the Food and Drug Administration (FDA) [1], and has been widely used in medicine due to its anti-oxidant [2,3,4], anti-inflammatory [5,6,7], wound-healing [8,9] and anti-bacterial [10,11] properties. Recent research has demonstrated that CUR has the ability to inhibit carcinogenesis in various cell lines, including breast, colon and gastric cancer cells, which has resulted in an increased interest as a promising anticancer drug [12,13,14,15]. However, CUR exhibits poor solubility in aqueous solutions, limiting its applications for cancer therapy [16,17,18].

It has been reported that after an oral administration of 2 g/kg of CUR in humans an extremely low serum concentration (0.006 ± 0.005 μg/mL) of CUR was observed after 1 h [19]. As a result, the bioavailability and anti-cancer efficiency of CUR is limited by its low solubility [16,17,18]. In order to improve the bioavailability, various nanocarriers have been used, including lipid-based nanoparticles [20,21,22,23,24], polymer nanoparticles [17,25,26,27,28,29,30] and inorganic nanoparticles [31]. The main advantages of the CUR loaded nanocarriers are their small size and large surface area, which enable them to pass through the cell membranes with an enhanced uptake efficiency [1,29]. Research has increasingly focused on the fabrication of biopolymer nanoparticles for CUR delivery, due to advantages of low cytotoxicity, excellent biocompatibility and biodegradability [29]. Two of the most commonly used biopolymers in medical applications are alginate and chitosan (CHI). Alginate is an anionic polysaccharide composed of (1–4)-linked β-d-mannuronate (M) and α-l-guluronate (G) residues, while CHI is a cationic polysaccharide composed of *N*-acetyl-β-d-glucosamine and β-d-glucosamine [32] and is positively charged below neutral pH, due to the protonation of amino groups [33]. Moreover, alginate based nanoparticles can be fabricated by simple processes, such as Ca^2+^, cross-linking or altering of pH [34]. CHI based nanoparticles can be prepared by via polyanion of tripolyphosphate (TPP) without introducing harsh cross-linking agents or organic solvents [35]. Electrostatic interactions between positive CHI chains and negative drugs, such as CUR, enable the retention of the drug in CHI based nanoparticles providing a prolonged drug release profile [36]. These advantages of alginate and CHI make them promising candidates as nanocarriers for drug delivery [37]. Various alginate or CHI based nanoparticles have been developed for the delivery of CUR [30,38,39,40,41].

Targeted delivery by magnetic nanoparticles (MNPs) has been reported as a promising strategy for cancer therapy with the advantages ranging from visualisation of the targeting process, rapid targeting and accumulation of drug carriers at the tumour sites via magnetic forces. The MNPs can be heated in a magnetic field to promote the drug release [42]. MNPs have been reported to have very low toxicity within the human body [43]. Their small size and large surface area make them suitable for polyelectrolyte layer-by-layer deposition. The incorporation of MNPs has shown the targeted delivery of drugs to tumour sites with the help of external magnetic fields [42]. For example, Mancarella et al. [44] developed layer-by-layer functionalized nanoparticles by coating MNPs with positively charged Poly-l-lysine and negatively charged Dextran. The layer-by-layer coating enabled a high loading efficiency of CUR into the particles, and the MNP cores promoted the uptake of CUR into SKOV-3 cells. In another example Pavlov et al. [45] prepared luciferase enzyme and plasmid DNA loaded particles with alternative layers of poly-l-arginine hydrochloride and dextran sulphate sodium salt. MNPs were incorporated into the particles and the resulting MNPs improved the delivery of enzymes and plasmids into 293T cells. In addition, MNPs could be efficiently navigated to cells with a magnet below the targeted tissue culture wells [45].

Exploiting the electrostatic properties of alginate and CHI, a layer-by-layer coating method can be employed to prepare multilayer alginate/CHI polyelectrolyte nanoparticles, allowing desired surface features to be engineered for specific applications [46]. By altering the number of layers deposited, it is possible to encapsulate a high payload of drugs and control the drug release rate [47,48,49,50]. In this paper, magnetic alginate/CHI layer-by-layer nanoparticles (MACPs) were fabricated for the delivery of CUR into MDA-MB-231 breast cancer cells and HDF cells.

## 2. Materials and Methods

### 2.1. Materials

Curcumin (C8069) was purchased from LKT Laboratories (Cambridge, UK). Paraformaldehyde (sc-253236A) was purchased from Chem Cruz^®^ (Dallas, TX, USA). Ca(OH)_2_ (21181) was purchased from Honeywell Fluka™ (Bucharest, Romania). Sodium alginate (W201502), Na_2_HPO_4_ (S7907), NaH_2_PO_4_ (S8282), ammonium hydroxide (221228), and DMSO (Dimethyl sulfoxide, D5879) were purchased from SIGMA-ALDRICH (Gillingham, UK). RPMI (Roswell Park Memorial Institute) 1640 Medium (BE 12-167F), PBS (Dulbecco’s Phosphate Buffered Saline, BE17-512F), Penicillin 5000 U/ml-Streptomycin 5000 U/mL (DE17-603E), l-Glutamine (17-605F) were purchased from Lonza^®^ (Manchester, UK). Chitosan (349051000), Iron (II) chloride tetrahydrate (44939), Iron (III) chloride hexahydrate (44944), NaHCO_3_ (A17005), Alexa Fluor^®^ 568 phalloidin (A12380), MTT (3-(4,5-Dimethylthiazol-2-yl)-2,5-Diphenyltetrazolium Bromide, M6494) assay, FBS (Fetal Bovine Serum, 10500064) and DAPI (4′,6-Diamidino-2-phenylindole dihydrochloride, D1306) were purchased from Thermo Fisher Scientific (Waltham, MA, USA). MDA-MB-231 cells (Human Caucasian Breast Adenocarcinoma cells) were purchased from ECACC (Salisbury, UK). Neonatal foreskin HDFs (Human Dermal Fibroblasts) were obtained from the Sheffield RNAi Screening Facility (Sheffield, UK).

### 2.2. Preparation of Nanoparticles

#### 2.2.1. Preparation of Fe_3_O_4_ Magnetic Nanoparticles

Fe_3_O_4_ MNPs were prepared by co-precipitation as previously described in Song et al. [27]. 4 g Iron (III) chloride hexahydrate and 4.5 g Iron (II) chloride tetrahydrate were each dissolved in 150 mL deionized (DI) water and degassed with nitrogen for 30 min to remove any dissolved oxygen. The solutions were then mixed in a 500 mL round-bottom flask and 15 mL of ammonium hydroxide was added under vigorous stirring under a nitrogen atmosphere at room temperature. The solution was then vigorously stirred for 2 h and the synthesised MNPs were collected with strong Neodymium magnets and washed several times with DI water until neutral pH. Finally, the magnetic nanoparticles were dried over night at room temperature and stored for future usage.

#### 2.2.2. Preparation of Magnetic Alginate Nanoparticles

Magnetic alginate nanoparticles (MAPs) were prepared based on the procedure described by Liu et al. [51] Briefly, 20 mL of ethanol and 10 mL of DI water was mixed into a beaker and 0.25 g of MNPs were suspended in the prepared mixture. Next, 40 mL of sodium alginate (SA) solution (20 mg/mL) was added into the beaker, upon which the mixture was sonicated for 10 min to allow full homogenous dispersion of MNPs in the suspension. The resulting suspension was vigorously stirred for 30 min at room temperature. Subsequently, 128 mL of Ca(OH)_2_ solution (0.74 mg/mL) was added into the suspension and stirred for 1 h before 16 mL of NaHCO_3_ solution (10 mg/mL) was added. The suspension was then stirred for a further 12 h at room temperature and the resulting MAPs were collected with strong Neodymium magnets, washing thoroughly with ethanol and water to remove any excess salts. Finally, purified MAPs were re-suspended in DI water before being used for the layer-by-layer coating process.

#### 2.2.3. Preparation of Magnetic Alginate/Chitosan Layer-by-Layer Nanoparticles

The preparation of magnetic alginate/chitosan layer-by-layer nanoparticles (MACPs) was based on the layer-by-layer self-assembly of SA and CHI on MAPs. Chitosan solution (10 mg/mL) was prepared in 1% (*v/v*) acetic acid aqueous solution. The first layer was deposited by adding 1 g of MAPs into 100 mL of the chitosan solution under vigorous stirring for 20 min at room temperature. The resulting MA/CHI particles were collected with a Neodymium magnet and the excess CHI was removed by washing the particles several times with DI water. The next SA layer was deposited by adding the previously prepared particles into 100 mL of SA solution (10 mg/mL) under vigorous stirring for 20 min thus forming MA/CHI/SA particles. For each layer the previously described purification process was used. Particles with more layers were fabricated by alternatively coating positively charged CHI and negatively charged SA on MACPs until the desired number of layers was reached.

### 2.3. Curcumin Loaded Magnetic Alginate/Chitosan Layer-by-Layer Nanoparticles 

20 mL of CUR solution (7.5 mg/mL in DMSO) was added into 30 mL of DI water to prepare the CUR mixture (3 mg/mL). Then 30 mg of MACPs were added into 20 mL of the CUR mixture and stirred for 24 h. The resulting CUR loaded magnetic alginate/chitosan layer-by-layer nanoparticles (CMACPs) were collected with a Neodymium magnet and washed three times with DI water. The supernatants were collected and analysed with a UV-Vis spectrometry (JENWAY 6715, Bibby Scientific, Stone, UK) to determine the concentration of residual CUR. The CMACPs were dispersed in 5 mL of DI water and the concentration of these particles was determined by weighing dried particles from 1 mL solution. Encapsulation and loading efficiencies were determined by Equation (1), where *EE%* is the Encapsulation Efficiency, *mP_CUR_* is the amount of CUR encapsulated in particles, and *m_intCUR_* is the amount of CUR initially added. In Equation (2), *LE%* is the Loading Efficiency and *N_P_* is the total amount of CUR loaded particles:

(1) $$EE{\%}_{\left(w/w\%\right)}=\frac{mP_{CUR}}{m_{\mathrm{int}CUR}}\times 100\%,$$

(2) $$LE{\%}_{\left(w/w\%\right)}=\frac{mP_{CUR}}{N_{P}}\times 100\%.$$

### 2.4. Release of CUR from CMACPs

5 mg of CMACPs were suspended in 5 mL PBS buffer (pH 7.4) in vials and incubated at 37 °C under constant agitation (200 rpm). The particles were collected with strong Neodymium magnets at pre-determined time points and the supernatants were carefully removed before re-dispersing the particles in 5 mL fresh PBS. The CUR concentrations of the supernatants were analysed using UV-Vis-spectrometry and the percentage of cumulative CUR release was plotted as a function of incubation time.

### 2.5. Particle Characterisation

#### 2.5.1. Nanoparticle Tracking and Zeta Potential Analysis

MACPs and CMACPs were dispersed in sodium phosphate solution (10 mM, pH 7) and injected into the scattering cell of an NTA (Nanoparticle Tracking Analysis, Nanosight LM10, Malvern, UK) where their motion was analysed and average size was calculated. The zeta potential measurement of particles was carried out by a Dynamic Light Scattering analyser (DLS, NanoBrook 90 plus Pals Particle size Analyzer, Brookhaven Instrument, Holtsville, NY, USA). Particles were washed several times and dispersed in DI water (pH 6.5–7.5) or 10 mM sodium phosphate buffer (pH 7) prior to zeta potential measurements.

#### 2.5.2. Fourier Transform Infrared Spectroscopy

Fourier transform infrared spectroscopy (FTIR) analysis was conducted with a Spectrum 100 spectrophotometer (PerkinElmer, Waltham, MA, USA). Particles were washed three times with DI water and dried in an oven at 60 °C for 24 h before being placed on the diamond attenuated total reflectance (ATR) accessory and compressed. The wavenumber region was set from 4000 to 600 cm^−1^ with a resolution of 1 cm^−1^. The spectral processing was conducted with IRPal 10 software.

#### 2.5.3. Atomic Force Microscopy Analysis

The aggregation size and morphology of particles was characterised using Atomic Force Microscopy (AFM, Dimension Icon with ScanAsyst, Bruker Corporation, Billerica, MA, USA). Particle suspensions were dropped on mica substrates and air dried before being placed on the sample stage. AFM measurements were conducted using ScanAsyst mode and SCANASYST-AIR tips (spring constant: 0.4 N/m, length: 115 μm, width: 25 μm, resonant frequency: 70 kHz) and data were analysed with NanoScope Analysis 1.5 software.

#### 2.5.4. Transmission Electron Microscopy Analysis

MACPs or CMACPs were dispersed in DI water and dropped onto copper TEM (transmission electron microscope) grids and incubated at room temperature for 30 s. Excess solution on the grids was then removed with filter paper, by gently dapping the edge and allowing excess liquid to be absorbed prior to TEM imaging (Tecnai G2 Spirit, FEI, Hillsboro, OR, USA). An acceleration voltage of 80 kV was used and images were recorded using a Gatan Orius SC1000B bottom mounted digital camera and analysed in Gatan Digital Micrograph software (version 3.9.1).

### 2.6. Cellular Uptake Assays

CUR medium solutions were prepared by adding CUR dissolved in DMSO (50 mg/mL) dropwise into media to obtain different final CUR concentrations (0.5, 1.5, 5, 15, 30 μg/mL). CMACPs were dispersed in media to reach the final CUR concentrations equivalent to those of CUR medium solutions. MDA-MB-231 and HDF cells were seeded onto 6 well plates at a density of 3 × 10^5^ cells per well and incubated overnight at 37 °C and 5% CO_2_. Then the media were replaced with 2 mL CUR or CMACPs medium solutions and incubated for a further 24 h. After this, the cells were harvested with trypsin and washed twice with PBS to remove any free CUR or CMACPs. The resulting cells were collected and analysed with an BD™ LSR II flow cytometer (BD Biosciences, USA) to investigate the cells CUR uptake.

### 2.7. Magnetically Targeted Delivery Assay

MDA-MB-231 cells were seeded in glass bottom dishes (Nunc™, diameter 35 mm, Thermo Fisher Scientific, Loughborough, UK) at a density of 3 × 10^5^ cells per dish and incubated overnight to allow attachment. Free CUR and CMACPs were added into cell cultures to reach a final CUR concentration of 5 μg/mL and incubated for 4 h. Magnetically targeted delivery was conducted by initially placing a Neodymium magnet under the dish for the first 15 min during incubation. Cells were then washed twice with PBS buffer and fixed with 4% paraformaldehyde. The fixed cells were stained with Alexa Fluor 568 Phalloidin and DAPI for 1 h. Confocal fluorescence images of cellular uptake of CUR were taken with an Inverted Zeiss LSM 510 NLO microscope (Zeiss, Oberkochen, Germany) and analysed with LSM Image Browser software version 4.2.0.121.

### 2.8. In Vitro Cytotoxicity Assay

The cytotoxicity of free CUR, MACPs, and CMACPs against MDA-MB-231 and HDF cells was investigated using the MTT assay. MDA-MB-231 or HDF cells were seeded into 96-well plates at a density of 5 × 10^3^ cells per well and incubated overnight at 37 °C and 5% CO_2_. The media were then removed and replaced with CUR medium solution, MACPs or CMACPs dispersions (CUR content in the particles is equivalent to the dosage of free CUR) to reach the final CUR concentrations of 0.5, 1.5, 5, 15, 30 μg/mL. After 48 h of incubation, 50 μL of 3 mg/mL MTT was added to each well and incubated at 37 °C for 3 h followed by removing the supernatants and adding 200 μL DMSO. A plate reader (FLUOstar galaxy, BMG LABTECH, Ortenberg, Germany) was used to measure the absorbance of each well, including control wells, containing only cells and medium at 570 nm. The relative cell viability was determined by comparing the absorbance with control wells.

## 3. Results and Discussion

### 3.1. Characterisation of Magnetic Alginate/Chitosan Layer-by-Layer Nanoparticles and Curcumin Loaded Magnetic Alginate/Chitosan Nanoparticles

#### 3.1.1. Zeta Potential, Size and FTIR Spectra

**Scheme 1:**
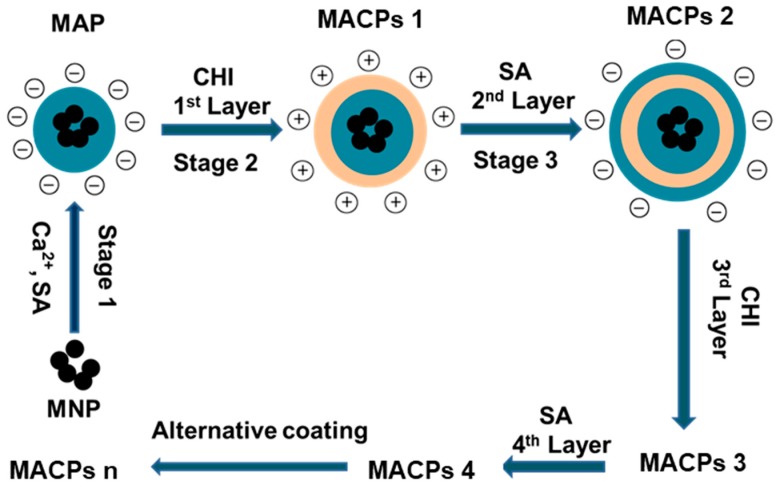
Schematic illustration for the preparation of MAPs and MACPs. MAPs were fabricated by coating cross-linked alginate on MNPs using Ca^2+^ as the cross-linker. MACPs were prepared by alternatively depositing CHI and sodium alginate (SA) on MAPs based on the electrostatic interaction between the two biopolymers. The alternative coating was repeated until MACPs with desired number of layers were obtained (Stage 2 and 3). MACPs 1, 2, 3, 4 and n represent CMACPs that possess 1, 2, 3, 4 and n layers of polymers coated on MNPs respectively.

As shown in Scheme 1, magnetic alginate particles (MAPs) were prepared via the self-assembly of alginate on the surface of MNPs in Ca^2+^ solution (Stage 1). Following this the formation of MACPs (Stage 2) was achieved by coating MAPs with CHI. Stage 3: Particles with 1 layer of SA and CHI were then coated with SA again. Stage 2 and 3 were then repeated consecutively for the desired number of layers.

**Figure 1:**
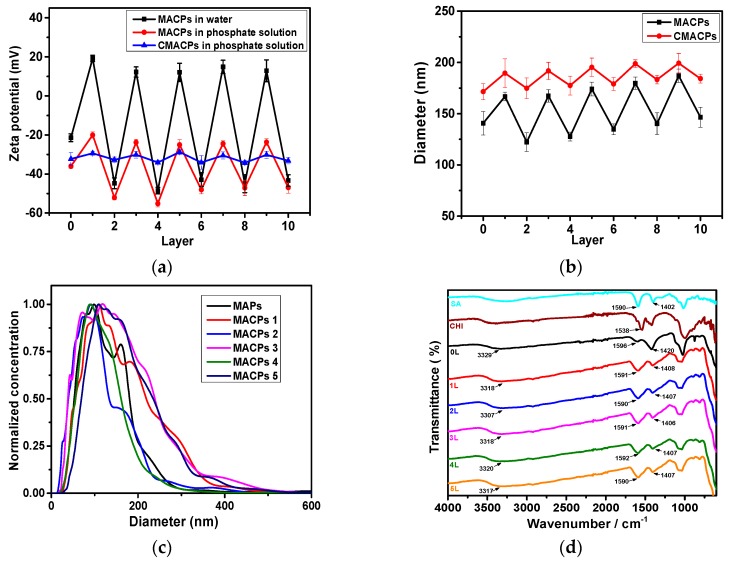
Zeta potential (**a**) and average diameter (**b**) of MACPs and CMACPs as a function of the number of coated layers. Zeta potential of particles was analysed in water (pH 7) and phosphate solution (10 mM, pH 7). Particle size was analysed in phosphate solution. Oscillations of zeta potential and average diameter were observed with the alternate coating of SA and CHI; (**c**) Size distribution of MAPs and MACPs with 1–5 layers of coated polymers; (**d**) FTIR spectra of MAPs and MACPs with the number of deposited polymer layers from 1 to 5. The results are shown in mean ± SD, n ≥ 3.

MAPs and MACPs with different number of layers were dispersed in DI water (pH was adjusted to 7 using HCl and NaOH) or sodium phosphate solution (pH 7) and their zeta potential was analysed using DLS. As shown in Figure 1a, the zeta potential of MAPs in water was −21.4 mV which was similar to the results of Ca^2+^ crosslinked alginate particles reported in previous literature [52,53]. The zeta potential of free SA in water has been reported as −50 mV [54]. The reason for the increased zeta potential of MAPs is due to crosslinking with Ca^2+^. The carboxyl groups contribute to the anionic charge of the alginate polymer [55]. Upon gelation, the negatively charged carboxyl groups from the G blocks in the alginate chains interact with Ca^2+^ ions and form an ‘egg-box’-like structure, reducing the density of free carboxyl groups, as well as the anionic charges [55]. Therefore, an increase of zeta potential was observed for MAPs. After deposition of the first layer (CHI), the zeta potential of the particles became positive (+19.2 mV) followed by a reduction to −44.8 mV after the negatively charged alginate polymer was coated as the second layer. Positive charges ranging from +12.1 to +19.2 mV were observed when CHI was coated as the outermost layer and negative charges ranging from −43 to −48.5 mV were associated with alginate coatings as the outermost layer. An oscillation of zeta potential was observed with the alternative deposition of SA and CHI, indicating the successful coating of the two polymers.

The zeta potential of particles in sodium phosphate solution (10 mM) is also shown in Figure 1a and exhibited a similar oscillation behaviour between coatings. However, negative surface charges were observed for MACPs (layer number: 1, 3, 5, 7, 9) with CHI outermost layer. This phenomenon is due to the adsorption of the phosphate anions onto the surface of the chitosan polymers. Phosphates contain small multivalent anions and therefore tend to interact or complex with cationic amino groups on CHI surfaces via electrostatic interactions [35]. Therefore, the phosphate anions adsorbed onto CHI layers increased the overall negative charge of the particles. Similar phenomena have been reported previously: For example, Swain et al. [56] analysed the zeta potential of CHI in both 1 and 10 mM NaCl and found that the zeta potential value decreased as the ionic strength of NaCl increased. The authors suggested the reduced surface charge was attributed to the adsorption of anions onto the surface of CHI polymers [56]. Further to this, Acevedo et al. [57] analysed the zeta potential of CHI (5 mg/mL) in water and NaCl solution (0.2 M) and reported zeta potential values of +72 mV in water and +33 mV in NaCl. It was suggested that this reduced absolute zeta potential value was governed by a compression of the electrical double layer, due to the electrostatic interactions with Cl^−^ in the solvent [57]. As shown in Figure 1a, the zeta potential of MACPs with alginate as the outermost layer in phosphate solution exhibited similar values as they were in water, which is in agreement with previously reported data [54]. On the other hand, MAPs exhibited a more negative zeta potential values in phosphate solution (−36 mV) compared to water (−21.4 mV). This is due to reaction between Ca^2+^ and phosphate ions to form insoluble calcium phosphate, which leads to the loss of Ca^2+^ and the increased density of free carboxyl groups thus resulting in an overall increased negative charge. The zeta potential behaviour of drug carriers can be utilised to control the drug loading and release processes. For example, cationic drug carriers, such as CHI nanoparticles, can be used to improve the loading of anionic drug molecules and prolong their release time based on electrostatic interactions between positive CHI and negative drug molecules [36]. In the case of MACPs, different numbers of polymer layers with different zeta potentials can potentially be used to improve drug loading.

The average diameter of MACPs was analysed by NTA, as shown in Figure 1b, where the diameter of MACPs was found to be 141 nm which increased to 167 nm after the first CHI layer coating. The size of particles increased gradually per bi-layer of SA/CHI and reached 187 nm for 9 layers. Size differences were observed depending on the outermost polymer of MACPs. For example, the average diameter of MACPs decreased from 167 (3 layers) to 128 nm (4 layers) and then increased to 174 nm after the deposition of CHI as the fifth polymer layer. This leaves a mean size difference of ~40 nm between CHI and SA. In addition, the size distribution of MAPs and MACPs (with 1–5 layers of coated polymers) is shown in Figure 1c. Broader size distributions were observed for particles with CHI as the outermost layer (MACPs 1, 3 and 5) compared to particles with alginate as the outermost layer (MAPs and MACPs 2 and 4). These results illustrate that particles with CHI outermost layers are larger and less condensed than particles with alginate as the outermost layer. This is most likely due to the different surface charges of the particles. For MACPs with CHI as the outermost layer, the zeta potential ranged from −20 to −25.1 mV in phosphate buffer solutions, while the zeta potential for alginate as the outermost layer ranged from −47 to −55.2 mV. Particles with absolute zeta potential values higher than 30 mV can be considered stable in solution [58]. Therefore, MACPs with alginate outermost layers were very stable in solution, due to their sufficient surface charges. On the other hand, particles with CHI as the outermost layer were less stable and more likely to aggregate in aqueous solutions forming larger particles.

The zeta potential of CMACPs in sodium phosphate solution at pH 7 is shown in Figure 1a. A significant change in surface charge was observed for particles after CUR loading. The zeta potential of free CUR sodium phosphate solution was −31.1 mV. In contrast, the zeta potentials of MAPs and MACPs with the outermost layer of alginate (layer number: 0, 2, 4, 6, 8, 10) was within the range from −36 to −55.5 mV (Figure 1a) while the zeta potentials of the particles with CHI outermost layer (layer number: 1, 3, 5, 7, 9) were around −20 mV. After CUR loading, the zeta potentials of CMACPs were found between −28.7 and −34.2 mV suggesting CUR was encapsulated in or adsorbed onto the particles, reducing the difference of the zeta potentials between MACPs with alginate and CHI outermost layers. Increased particle sizes (between 172–199 nm) were observed for CMACPs (Figure 1b) compared to MACPs (122–187 nm), which can be attributed to the loading of CUR. For CMACPs with the same outermost polymer (SA or CHI), the average diameter of particles increased as the number of layers increased, due to the fact that larger polymer matrixes encapsulate more CUR molecules. Larger particle size was observed for CMACPs with the outermost polymer being CHI for the same reason as described above. The resulting particle sizes are optimal for drug delivery applications [59]. The chemical compositions and interactions between different polymer layers were analysed by FTIR, as shown in Figure 1d. For SA, the peaks at 1590 cm^−1^ and 1402 cm^−1^ were characteristic for the asymmetric stretching and symmetric stretching of -COOH groups respectively [60,61]. In MAPs and MACPs with the number of layers (1, 2, 3, 4 and 5), these two peaks moved towards higher wavenumbers, indicating the formation of Ca^2+^-alginate ionic cross-linking [62]. This is because of the changes in charge density, atomic radius and atomic weight of the cations [62,63]. For CHI, the peak at 1538 cm^-1^ was characteristic of -NH_2_ bonds [64]. The presence of CHI in the MACPs was also confirmed by comparing the curves of MAPs (0 L) and MACPs (1–5 L). The broad characteristic adsorption at 3329 cm^−1^ for −OH groups in MAPs shifted to a lower frequency for MACPs (1–5 L), demonstrating the superposition of amine N–H stretching in chitosan and −OH groups of alginate and chitosan [64,65]. The peak at 1596 cm^−1^ in MAPs became broader, and moved to a lower frequency for MACPs, which can be attributed to the overlapped −NH_2_ stretching from chitosan and −COOH stretching from alginate [66]. These results confirmed that CHI and alginate chains were successfully incorporated into the MACPs.

#### 3.1.2. Morphology Characterisation

**Figure 2:**
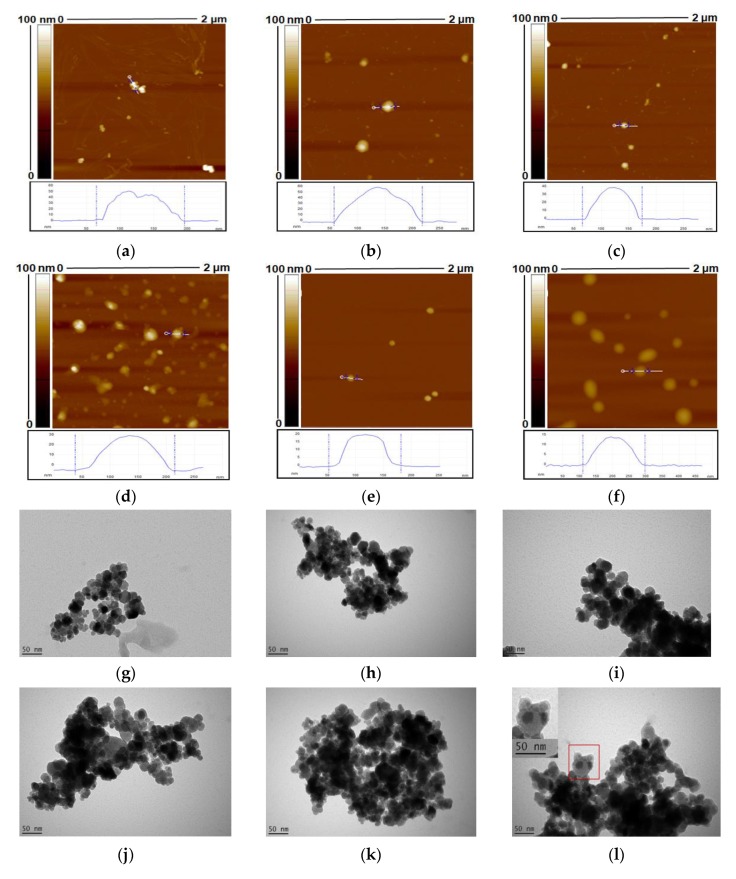
Atomic force microscopy (AFM) and transmission electron microscope (TEM) images of MAPs (**a**,**g**) and MACPs with the layer number of 1 (**b**,**h**), 4 (**c**,**i**), 5 (**d**,**j**), 8 (**e**,**k**) and 9 (**f**,**l**). Smaller particles were observed for MACPs with SA as the outermost layer.

The size distribution and morphology of MAPs and MACPs were also analysed by AFM and TEM (Figure 2). As shown in Figure 2a, the particles exhibited heights of 50 nm with bottom diameters around 140 nm, which is due to (1) The tip-sample convolution effect, leading to the lateral broadening of surface protrusions [67]; and (2) The aggregation of small MAPs, flattened on mica substrates, leading to the expanded bottom diameter. MACPs (Figure 2b,d,f) with CHI as the outermost layer showed larger bottom diameters than those (Figure 2c,e) with SA as the outermost layer, suggesting larger aggregation occurred for particles with CHI as the outermost layer. This is also due to the fact that the mica surface is negatively charged, therefore particles with positive net charges attach tightly and become more fat and flat. This result is consistent with the data obtained from NTA, confirming that MACPs with CHI as the outermost layer are loosely packed due to their lower net surface charges. Particles with alginate as the outermost layer are negatively charged and expected to be less attached therefore are more roundly shaped. Cross section AFM images indicated that MAPs (Figure 2a) possess rough topography, which were attributed to the aggregation of small solid MAPs. After alternate coating with SA and CHI, the resultant MACPs (Figure 2b–f) exhibited more spherical shapes and smooth surfaces, which further confirmed the successful coating of both polymers. For MACPs, inter-digitation among the adjacent CHI/SA layers were constructed due to the existence of surface charges. Therefore, after the removal of excess polymers, stable films were formed by soft polymers of CHI and SA leading to the smooth surface of MACPs.

Characterisation of MAPs and MACPs via TEM revealed magnetic cores (dark areas) surrounded by polymer shells, which were the chitosan and alginate layers, as shown in Figure 2. The inset in Figure 2i. is an enlarged section of the image clearly showing magnetic core particles and the surrounding CHI/SA layers. It can be observed from Figure 2 that particle aggregates were formed on the TEM grids. This is most likely due to the aggregation of particles during the drying process. In solutions, the magnetic core-shall particles are stabilized by the charged polymer layers (chitosan and alginate) and the aggregation degree of particles is related to the balance between the repulsive electrostatic interactions and the attractive interactions. During the drying process, the concentration of particles increased, which will increase the probability of particle collisions and enhance the attractive interactions, leading to promoted aggregation of particles.

**Figure 3:**
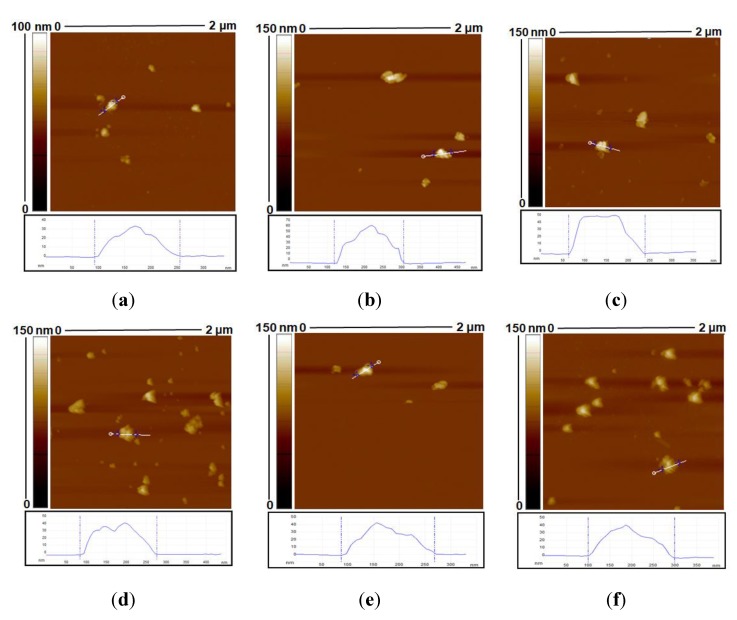
AFM images of Curcumin (CUR)-loaded MAPs (**a**) and CUR loaded MACPs (CMACPs) with the layer number of 1 (**b**), 4 (**c**), 5 (**d**), 8 (**e**) and 9 (**f**).

AFM was also used to investigate the morphology of CMACPs. As shown in Figure 3a, the bottom diameter of CUR loaded MAPs was ~150 nm and increased layer by layer, reaching a maximum of ~200 nm for 9 layers (Figure 3f), which is in agreement with the size tendency analysed by NTA, as shown in Figure 1b. However, rougher surface morphology was observed for CMACPs compared to MACPs, as shown in Figure 2. CUR is known as a highly hydrophobic drug [68,69,70] and the loading of CUR into MACPs increased the hydrophobicity of the particles. This meant that the particles rapidly dehydrated during AFM analysis and exhibited rough surfaces, indicating the successful loading of CUR into MACPs.

### 3.2. Loading and Release of Curcumin

**Figure 4:**
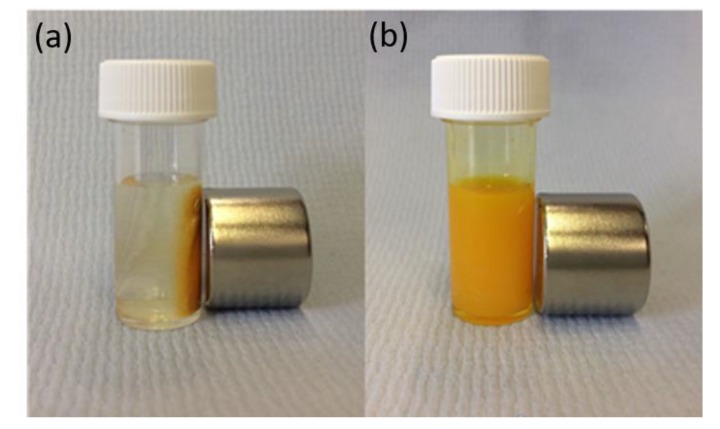
CMACPs were prepared by suspending MACPs in CUR water/DMSO solution and incubating for 24 h under stirring. The resulting CMACPs can be rapidly collected by a Neodymium magnet (pull force 25 Kg, 25.4 mm diameter × 30 mm thick) within 1 min (**a**). The free CUR in solution on the other hand was not affected by the magnetic force (**b**).

CUR loaded MACPs were prepared by dispersing MACPs in CUR water/DMSO solution and incubated under stirring for 24 h. The addition of DMSO increases CUR solubility and its permeability into MACPs. The volume ratio of water:DMSO was 3:2, resulting in a homogeneous CUR solution (3 mg/mL) without sedimentation during the 24 h of incubation [71]. Figure 4a clearly demonstrates that the resultant CMACPs can be collected with a Neodymium magnet while CUR itself in the bulk solution was not affected by the magnetic field. Therefore, MACPs can be potentially used for targeted delivery of CUR via external magnetic fields.

**Figure 5:**
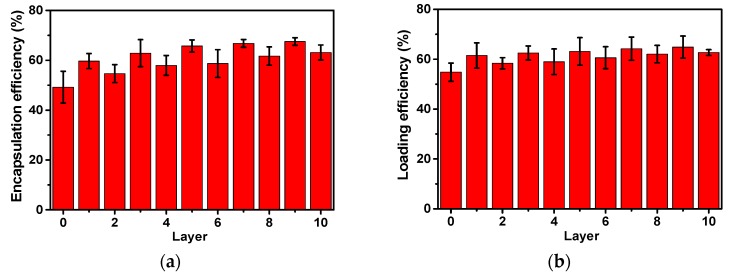

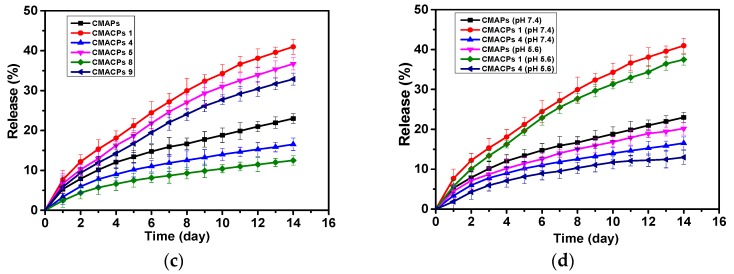
The drug loading and release profiles of the CMAPs and CMACPs are affected by the type of outermost polymers and the number of layers. Encapsulation efficiency of CMAPs and CMACPs (**a**). Curcumin loading efficiency into CMAPs and CMACPs (**b**). Curcumin release profile of CMAPs and CMACPs with different layers (1, 4, 5, 8, 9) of deposited polymers (**c**). Curcumin release profile of CMAPs and CMACPs (layer number: 1 and 4) in PBS buffer at pH 7.4 and pH 5.6 (**d**). The results are shown in mean ± SD, n = 3.

Encapsulation efficiency results reveal that 49.2% of CUR was loaded into CMAPs and the encapsulation efficiency increased to 67.5% with increasing number of layers of CHI and SA (see Figure 5a). For particles with the same type of polymer (CHI or SA) on the outermost surface an increased trend of encapsulation efficiency was observed with increasing layer number. However, as illustrated in Figure 5a, a higher encapsulation efficiency of CUR was observed for CMACPs with CHI as the outermost layer (layer number: 1, 3, 5, 7 and 9) compared to SA as the outermost layer (layer number: 2, 4, 6, 8, 10). The most feasible explanation for this is the different surface charges between the particle outermost layers. As illustrated in Figure 1a, CHI layers exhibited positive surface charges in water while SA layers exhibited negative surface charges. Therefore, the negatively charged CUR molecules are adsorbed on the surface of the CHI layer more readily, facilitating a more efficient and faster encapsulation into polymer matrix. On the other hand, SA layer was negatively charged in water, and thus CUR molecules were less readily to interact with the particle surface, due to the electrostatic repulsion. Moreover, larger amount of CUR molecules were expected on the surface of CHI layer, which also led to a higher encapsulation efficiency. The same hypothesis can be used to explain the loading efficiency of CMAPs and CMACPs. As shown in Figure 5b, a higher loading efficiency of CUR was observed for MACPs with CHI as the outermost layer to those with SA as the outermost layer. Higher loading efficiencies from 54.8% to 64.9% were achieved for CMAPs and CMACPs compared to many previously reported CUR loaded CHI and alginate based particles [25,28,38] with loading efficiencies from 2.7% to 48%, suggesting CMAPs and CMACPs possess great potential loading CUR into nanoparticles for drug delivery applications.

In order to investigate the release profile of CMAPs and CMACPs, 5 mg of particles were dispersed in 5 mL PBS buffer at pH 7.4 and incubated at 37 °C under shaking (200 rpm) at predetermined time intervals. The particles were collected with strong Neodymium magnets and the CUR concentration in supernatants were analysed to obtain the accumulative CUR release profiles. As shown in Figure 5c, for all particles, CUR was released more rapidly within the first two days and then released slower, but at more sustained rates in PBS for up to 2 weeks. The faster release in the first two days is most likely due to the loss of weakly adsorbed CUR molecules on the surface of the particles or in the polymer matrix close to particle surfaces. The sustained CUR release after the first two days was then dependent on the diffusion of CUR molecules through the polymer matrixes into the bulk PBS solution, thus exhibiting a slower and more sustained release pattern. No significant burst release was observed during the whole release process [26,72]. CMACPs with CHI as the outermost layer exhibited a faster release rate of CUR than CMAPs and CMACPs with SA as the outermost layer (CMACPs 4 and 8). As mentioned previously in Section 3.3.1, CHI is positively charged in water, but negatively in phosphate solution, due to the adsorption of negative phosphate ions [35], therefore more negatively charged CUR molecules are adsorbed onto the CHI layer surface when these particles were dispersed in CUR water/DMSO solution than on alginate, as illustrated in Figure 5a,b. When CMACPs with layer numbers of 1, 5 and 9 were dispersed in PBS, CUR molecules were released from the particle surface, due to CUR and phosphate ions competing for free amino groups on the CHI chains, this in turn facilitates the loss of CUR molecules from the inner layers to the surface via a concentration gradient driven diffusion. Thus, faster CUR release rates were observed for CMACPs with layer numbers of 1, 5 and 9. On the other hand, CMAPs and CMACPs with layer numbers of 4 and 8, where alginate was deposited as the outermost layer, showed similar negative surface charges in water and phosphate solutions, less CUR molecules were adsorbed during the loading process and thus the drug release rate was not promoted in phosphate solutions. For particles with the same type of polymer, reduced release rates were observed as the number of layers increased (release rate: CMACPs 1 > CMACPs 5 > CMACPs 9, CMAPs > CMACPs 4 > CMACPs 8). By increasing the number of polymer layers it was more difficult for the innermost CUR to permeate out. This is in agreement with previously reported data [47,48,49], where Chai et al. [47] fabricated doxorubicin loaded poly (lactic-co-glycolic acid) nanoparticles and layer-by-layer coated the particles with CHI and alginate. After comparing the drug release rates of uncoated and polymer coated particles, the authors found that with the CHI/SA coating the initial drug burst release was reduced from 55.1% to 5.8% and the overall drug release rate was also reduced. In another example Haidar et al. [48] prepared bovine serum albumin loaded liposomes and layer-by-layer coated them with CHI/SA. The polymer coated liposomes showed a reduced albumin release rate to the uncoated ones. Further to this Zhou et al. [49] developed polyethyleneimine coated PLGA nanoparticles and reported that the drug release rate reduced with increasing layers.

In order to investigate the drug release profile of CMAPs and CMACPs at different pH, CMAPs, CMACPs with 1 and 4 layers were dispersed in PBS with pH 7.4 and pH 5.6. As illustrated in Figure 5d, the release pattern of CUR from particles at pH 5.6 was similar to those at pH 7.4, but slightly slower. In 14 days, about 23%, 41% and 17% of CUR were released from CMAPs, CMACPs 1 and CMACPs 4 at pH 7.4 respectively, while about 20%, 37% and 13% of CUR were released at pH 5.6. The data are in agreement with previously published work by Martins et al. [41] who investigated the release profiles of CUR from N-trimethyl chitosan/alginate complexes at pH 7.4 and pH 1.2 and reported that 80% of CUR was released at pH 7.4 within the first hour while only 22% of CUR was released at pH 1.2. The pH dependent release pattern can be attributed to the altered surface charge and hydrophobicity of polymers at different pH values.

### 3.3. Cellular Uptake Assays

#### 3.3.1. CUR Uptake Assay by Flow Cytometry

To investigate the cellular uptake kinetics of CMAPs and CMACPs, MDA-MB-231 breast cancer cells and HDF cells were incubated with free CUR and particles containing equivalent amounts of CUR for 24 h. Except for skin cancers, breast cancer is most commonly diagnosed among US women and the second leading cause of cancer death among women [73]. Therefore, MDA-MB-231 breast cancer cells were selected as a cancer model and HDF cells were selected as a non-cancer model to investigate the uptake effect of the drug loaded particles.

**Figure 6:**
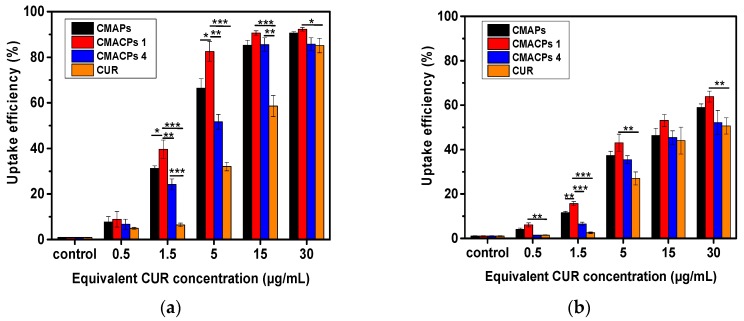
Cellular uptake analysis of CUR in MDA-MB-231 cells (**a**) and HDF cells (**b**) after incubation with CMAPs, CMACPs 1 and 4 and free CUR for 24 h. The CUR uptake was analysed by flow cytometry. The dosages of CUR in particles were equivalent to the amounts of free CUR used. CMACPs 1 and 4 represent CMACPs that possess 1 and 4 layers of polymers coated on MNPs respectively. The results are shown in mean ± SD, n = 3. The statistical significance is expressed as *** *p* < 0.001, ** *p* < 0.01, * *p* <0.05.

The cells were analysed by flow cytometry to determine the cellular uptake of CUR by different cells. As shown in Figure 6a, it is evident that the percentage of MDA-MB-231 cells taking up CUR was dose dependent. Significant higher uptake efficiency was observed for MDA-MB-231 cells treated with CMAPs and CMACPs than those treated with free CUR, indicating that the nanoparticles facilitate the CUR uptake. This is in agreement with previously reported data [1,17,74,75,76]. Here, at a CUR concentration of 1.5 μg/mL, an uptake efficiency of 31.2%, 39.6% and 24.2% was observed for MDA-MB-231 cells treated with CMAPs, CMACPs 1 and 4 respectively, which is 3–6 fold higher than the uptake efficiency of cells treated with free CUR. The increased CUR uptake of CMAPs can be attributed to the fact that the CUR loaded nanoparticles were more easily taken up by cells via endocytosis, due to their smaller size (172–199 nm), while highly hydrophobic CUR was insoluble in aqueous solutions forming larger aggregates, which are more difficult to be internalised by cells [75,77]. The order of uptake efficiency of free CUR and CUR loaded particles was CMACPs 1 > CMAPs >CMACPs 4 > free CUR. Particles with CHI as the outermost layer exhibited the highest uptake efficiency (39.6%). This result is in agreement with work by Zhou et al. [46], who compared the uptake efficiency of CHI/alginate coated poly (lactide-co-glycolide) nanoparticles and observed the uptake efficiency of particles with CHI as the outermost layer was the highest. The promoted CUR uptake by the CHI surface is because of the protonated amino groups, which interact with the negatively charged cell surfaces and promote the accumulation of CMACPs 1 onto the surface of MDA-MB-231 cells. Figure 6b shows the CUR uptake efficiency for HDF cells after being treated with free CUR and CUR loaded particles. HDF cells exhibited a similar uptake pattern, but an overall much lower CUR uptake efficiency compared to MDA-MB-231 cells, suggesting CMAPs and CMACPs target cancer cells rather than normal cells. This is in agreement with many previously published reports [28,78,79,80] as cancer cells possess a higher metabolic activity than normal cells and their surfaces overexpress various receptors that in turn increase the available binding sites promoting a higher uptake efficiency of particles and drugs [81,82,83].

#### 3.3.2. Magnetically Targeted Delivery Assay

**Figure 7:**
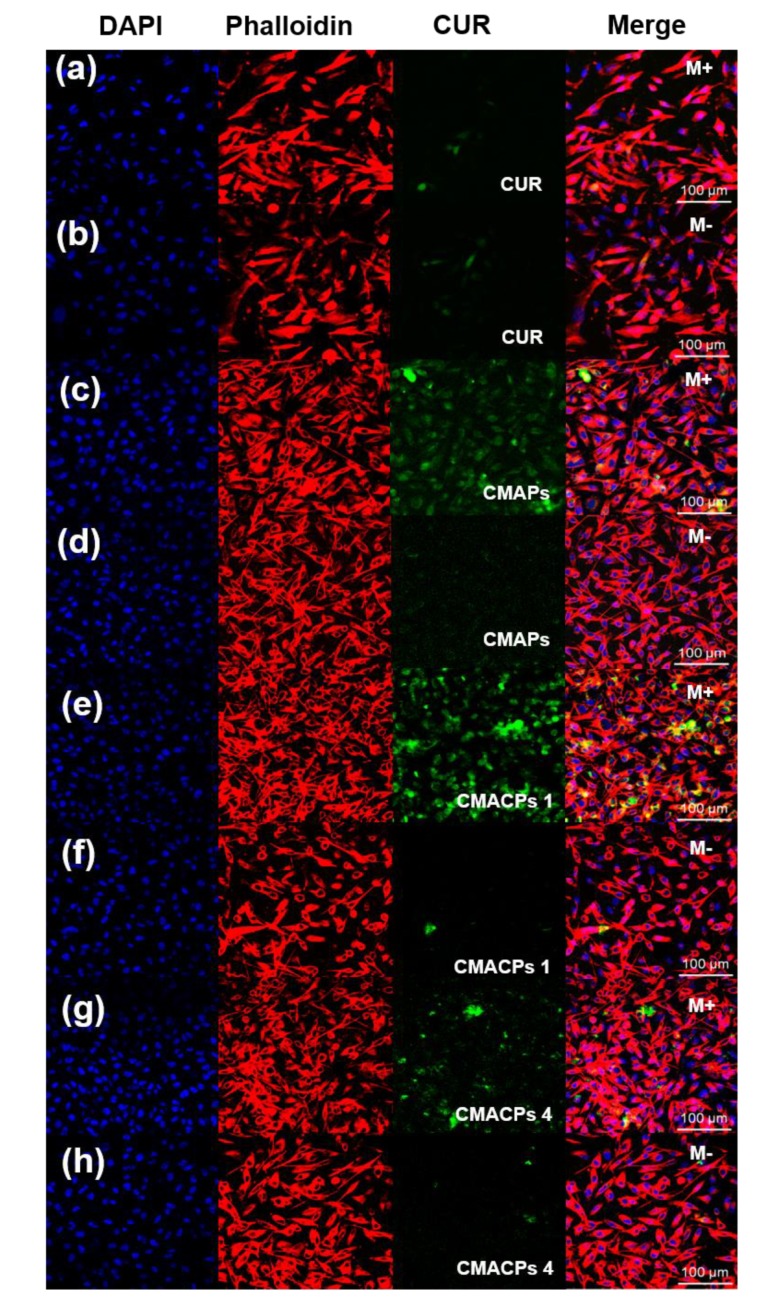
Confocal fluorescence microscopy images of MDA-MB-231 cells incubated with free CUR (**a**,**b**), CMAPs (**c**,**d**), CMACPs 1 (**e**,**f**) and CMACPs 4 (**g**,**h**). CMACPs 1 and 4 represent CMACPs that possess 1 and 4 layers of polymers coated on MNPs respectively. CUR loaded particles contained equivalent amount of drug as free CUR (5 μg/mL). Cell nucleus and cytoskeleton were stained with DAPI (blue) and Alexa Fluor 568 Phalloidin (red). M+ denotes the images taken within the magnet affected area and M- denotes the images taken outside of the magnet-affected target area.

To investigate the potential of CMAPs and CMACPs for magnetically targeted delivery of CUR, free CUR, CMAPs and CMACPs were incubated with MDA-MB-231 cells in glass bottom dishes. A Neodymium magnet was placed under the target area of the dish to allow for the accumulation of magnetic nanoparticles within the selected area. Taking advantage of the auto-fluorescent property of CUR, the confocal fluorescence images show the cellular uptake of CUR in and out of the target (magnet-affected) areas. The overall fluorescent intensity of MDA-MB-231 cells within the target areas that were treated with free CUR (Figure 7a) was lower than cells treated with CUR loaded nanoparticles (Figure 7c,e,g), suggesting CMAPs and CMACPs have enhanced the uptake of CUR. This result is consistent with the uptake efficiency shown in Figure 6. No obvious difference in fluorescent intensities was observed between Figure 7a and Figure 7b, showing that the free CUR uptake is not affected by the presence of magnetic forces. In contrast, cells treated with CMAPs (Figure 7c), CMACPs 1 (Figure 7e) and CMACPs 4 (Figure 7g) within the magnet-affected area showed significant increase of fluorescent intensity compared to the cells treated with same particles, but outside of the target (magnet-affected) areas (Figure 7d,f,h). The enhanced CUR uptake in the target areas was attributed to the accumulation of CUR loaded magnetic nanoparticles to the target areas driven by the magnetic force. On the other hand, the local concentration of CUR loaded magnetic particles outside the target areas was significantly lower, thus showing much lower fluorescent intensities. This result clearly shows that CMAPs and CMACPs have the potential for targeted drug delivery.

### 3.4. In Vitro Cytotoxicity Assay

**Figure 8:**
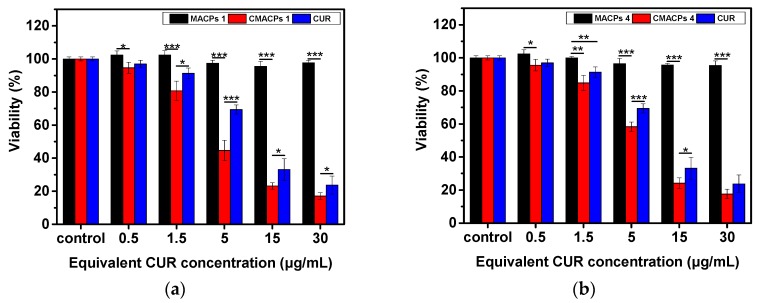

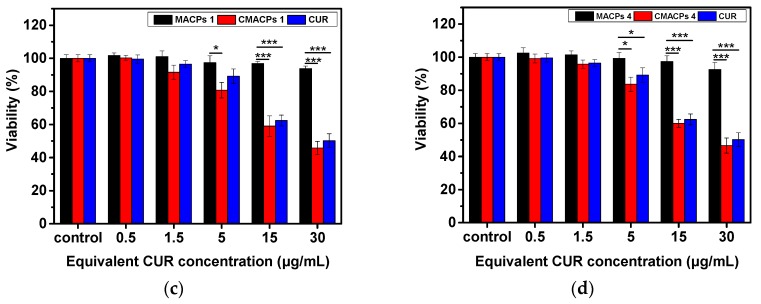
The in vitro MTT assay suggested CMACPs exhibited significantly higher cytotoxicity towards MDA-MB-231 breast cancer cells than to HDF cells. MDA-MB-231 cells (**a**,**b**) and HDF cells (**c**,**d**) were treated with free CUR, CMACPs 1, CMACPs 4 and blank MACPs 1, MACPs 4 for 48 h. CMACPs 1 and 4 (or MACPs 1 and 4) represent CMACPs (or MACPs) that possess 1 and 4 layers of polymers, respectively. The amounts of CUR in CUR loaded nanoparticles were equivalent to the amounts of free CUR (0.5–30 μg/mL), respectively. The results are shown in mean ± SD, n ≥ 3. The statistical significance is expressed as *** *p* < 0.001, ** *p* < 0.01, * *p* <0.05.

In order to investigate the cytotoxicity of CMACPs towards cancer and normal cells, MDA-MB-231 breast cancer cells and HDF cells were incubated with free CUR, CMACPs 1, CMACPs 4 and blank MACPs for 48 h. The in vitro MTT assay results are shown in Figure 8. It can be observed in Figure 8a,b that both free CUR and CMACPs exhibited increased cytotoxicity towards MDA-MB-231 cells with increasing CUR concentration, which was in agreement with previously reported data that CUR possesses dose dependent cytotoxicity towards cancer cells [1,17,74,75,76]. Both CMACPs 1 and 4 showed enhanced cytotoxicity towards MDA-MB-231 cells compared to free CUR, which can be attributed to an enhanced uptake of CMACPs. As discussed in Section 3.3.1, the uptake efficiency of MDA-MB-231 cells treated with CMACPs was 3–6-fold greater than those treated with free CUR. It is expected that the sustained release of CUR from internalised CMACPs maintained a high CUR concentration within the MDA-MB-231 cells, thus leading to lower viability of cells. CMACPs 1 were observed to exhibit higher cytotoxicity towards MDA-MB-231 cells than CMACPs 4, which was due to a higher CUR release rate (Figure 5c) and uptake efficiency (Figure 6a). As shown in Figure 8c,d, the viability of HDF cells after treatment with free CUR and CMACPs was reduced at higher CUR concentrations (>15 μg/mL), but a high viability was observed in contrast to MDA-MB-231 cancer cells at CUR concentrations below 15 μg/mL. One of the major accepted theories is that cancer cells possess lower glutathione levels than normal cells, due to their reprogrammed metabolic pathways [29]. Depletion of glutathione, which is important for the sensitivity of cells to CUR can lead to the enhancement of CUR sensitivity of cancer cells [29,78,84]. In addition, most cancer cells, but not normal cells, express constitutively active NF-KB that mediates their survival. CUR can suppress NF-κB regulated gene products, thus suppressing the proliferation of cancer cells [78,85]. High viabilities were observed for both MDA-MB-231 cells and HDF cells after they were treated with blank MACPs, demonstrating that MACPs are non-toxic towards these cells.

## 4. Conclusions

MACPs were prepared by a layer-by-layer coating of CHI and SA onto the surface of Fe_3_O_4_ nanoparticles. The successful coating of CHI and SA was confirmed by the zeta potential and FTIR spectral measurements of the particles. Incorporation of CUR was confirmed by a change in surface charge and morphology of CMACPs, while the mean diameter of CMACPs was lower than 200 nm and within the optimum size range for drug delivery applications. In vitro drug release profiles illustrated the sustained release of CUR from CMACPs and indicated that it was possible to control the release rate by altering the outermost polymer (CHI or SA), as well as by changing the number of layers. More biopolymer layers resulted in a slower CUR drug release profile, where CHI as the outermost layer showed a faster CUR release. Confocal fluorescence microscopic images confirmed the successful internalisation of CUR into MDA-MB-231 breast cancer cells and indicated that rapid and targeted delivery of CUR could be achieved in the presence of an external magnetic field. FACS analysis indicated the CMACPs mediated uptake of CUR by MDA-MB-231 cells was 3–6 fold greater than that of free CUR. MDA-MB-231 cancer cells showed a significantly higher uptake efficiency of CUR than that of HDF normal cells after being treated with CMACPs. The MTT assay indicated that CMACPs exhibited a significantly higher cytotoxicity towards MDA-MB-231 cancer cells than towards HDF cells. In summary, the sustained release profiles, enhanced uptake efficiency and strong cytotoxicity to cancer cells, as well as the potential for targeted delivery make MACPs a promising candidate for anti-cancer drug delivery.

## Abbreviations

CUR, curcumin; CHI, chitosan; MNPs, magnetic nanoparticles; MAPs, Magnetic alginate nanoparticles; MACPs, magnetic alginate/CHI layer-by-layer nanoparticles; CMACPs, Curcumin loaded magnetic alginate/chitosan layer-by-layer nanoparticles; CMACPs 1 and 4, Curcumin loaded magnetic alginate/chitosan layer-by-layer nanoparticles with 1 and 4 layers of CHI/SA coating; HDF, Human Dermal Fibroblasts.
